# Mechanisms of Learning in Adults With ADHD During an Ecologically-Valid Visual Discrimination Task

**DOI:** 10.1177/10870547251356744

**Published:** 2025-08-07

**Authors:** Elizaveta Kuznetsova, Tuisku Tammi, Natalia Postnova, Jussi Palomäki, Benjamin Ultan Cowley

**Affiliations:** 1 Faculty of Educational Sciences, University of Helsinki, Helsinki, Finland; 2 Cognitive Science, Department of Digital Humanities, Faulty of Arts, University of Helsinki, Helsinki, Finland; 3 Health and Well-Being Promotion Unit, Finnish Institute for Health and Welfare, Helsinki, Finland

**Keywords:** ADHD, learning, gestalt contours, Kanizsa, ERP, visual attention, 2AFC

## Abstract

**Objective:**

Learning unfolds in distinct stages—acquisition, consolidation, and maintenance—shaped by cognitive mechanisms such as saliency processing, interference control, and sustained attention. ADHD in adults is associated with deficits in these cognitive processes, which in turn might lead to learning difficulties.

**Method:**

Using a novel protocol that incorporates a visual attention task with gestalt-image targets and primer distractors, we investigated these cognitive mechanisms across different stages of learning in 53 adults diagnosed with ADHD and 18 neurotypical Controls.

**Results:**

Our findings reveal that adults with ADHD exhibit reduced neural activations in the occipital and parietal areas, indicating diminished bottom-up visual processing and challenges in handling distractions. Nevertheless, individuals with ADHD demonstrate increased frontal activity in the late stages of visual processing, suggesting compensatory mechanisms employed by the group. Behaviorally, both groups achieve comparable performance, though ADHD participants do so at the expense of greater variability and attentional lapses. Furthermore, while Controls reach the plateau already after the acquisition phase, the ADHD group is gradually improving its performance throughout the experiment.

**Conclusion:**

These findings demonstrate that adults with ADHD can acquire and retain new skills but do so through different—and usually more effortful—pathways. By mapping neural and behavioral dynamics onto learning stages, this study offers a more nuanced framework for learning in ADHD and supports the development of phase-specific intervention strategies.

## Introduction

Learning in humans is usually defined as the process by which a person acquires knowledge, improves skills, and adapts behaviors based on new information or experiences. It is a cornerstone of individual development and societal progress. Across disciplines, learning is typically parsed into three key stages: acquisition, consolidation, and maintenance (Fitts & Posner, 1967; Karni & Sagi, 1993; McClelland et al., 1995). Acquisition is the phase when individuals first encounter and acquire new knowledge or skills. It involves exposure and practice, and leads to initial neuroplastic connections, and memory formation related to the learned material. Consolidation is an intermediate stage, during which the newly acquired information becomes more stable and is integrated into existing knowledge structures. Finally, the maintenance stage occurs after consolidation and implies obtaining higher reliability of performance, especially in the face of distraction or ambiguity of the stimulus.

ADHD is a neurobiological condition that affects behavior and interferes with how individuals access and use their abilities to learn. Although early work focused primarily on children (Barkley, 1997; DuPaul & Volpe, 2009), growing evidence demonstrates that adults with ADHD also exhibit disrupted learning, particularly when tasks require sustained attention, feedback processing, and distraction management (Blomberg et al., 2022; Mohamed et al., 2021). Nevertheless, adults with ADHD may perform comparably to neurotypical individuals when tasks are intrinsically engaging or provide consistent, salient cues (Aster et al., 2024; Carlson et al., 2002). This reinforces the idea that learning outcomes in ADHD are not uniformly impaired but instead reflect interactions between core cognitive mechanisms such as saliency processing, inhibition of interference, and sustained attention. In the present study, we focus on three such mechanisms and link them to specific learning phases where they are most critically important: acquisition, consolidation, and maintenance, respectively.

Saliency refers to the quality of being particularly noticeable to the senses. Saliency processing is essential during the initial stages of learning as it allows the learner to quickly identify which aspects of the environment are most relevant to achieving the desired outcome. Research shows that adults with ADHD show altered neural responses to salient cues. For example, fMRI data indicate weaker functional segregation between the salience network and the default-mode network (Blomberg et al., 2022), leading to inefficient filtering of task-relevant input. This corresponds with evidence of lower dopamine levels in ADHD patients (Krause, 2008), as dopamine is known to modulate the attentional response to saliency (Volkow et al., 2004).

Inhibition of interference refers to the ability to suppress irrelevant or distracting information, preventing it from disrupting the learning and memory consolidation processes. Barkley (1997) claimed that poor behavioral inhibition, which is associated with the meso-cortical branch of the dopamine system projecting into the prefrontal cortex, is the central deficiency in ADHD and argued that the inhibitory deficits are responsible for secondary deficiencies in other executive functions. More recent EEG and behavioral studies in ADHD adults support this view, showing increased cross-modal interference—such as heightened visual cortex activity during auditory tasks—indicating a failure to gate irrelevant sensory input (Schramm et al., 2023). These deficits not only affect in-the-moment task accuracy but also hinder effective consolidation, as distraction during early encoding or rehearsal impairs memory stabilization (Fernandes & Moscovitch, 2000).

Learning to maintain can be largely affected by ability to sustain attention. Sustained attention is continuous and self-directed awareness of a subset of stimuli, that occurs when responding to approximately uniform task stimulation (Robertson et al., 1997; Unsworth & Robison, 2020). The ability to sustain attention involves resisting neuro-energetic fatigue and distraction, both of which have been implicated in hypotheses of ADHD and reflected in patients’ EEG signal (Gumenyuk et al., 2005; Killeen et al., 2013; Russell et al., 2006). Besides, empirical work using continuous performance paradigms reveals greater intra-individual variability in response times and degraded alerting signals (Xiang et al., 2024).

While saliency processing, inhibition of interference and sustained attention have each been well-studied in isolation, how they combine in ADHD is under-studied, despite this combination being exactly what is required to learn and perform complex naturalistic tasks. To address this gap, we have constructed a protocol to probe the interplay between these cognitive processes. Primed Subjective-Illusory-Contour Attention Task (PSICAT) uses gestalt-image targets to probe saliency, congruent and incongruent primer-distractors to probe inhibition and runs across 550 trials to probe sustained attention.

In this study we report results on the following research questions (RQs):

**RQ1**: How do ADHD and Control groups differ in learning acquisition, and is that moderated by the mechanisms of saliency processing?
**RQ2**: How do the groups differ in learning consolidation, and how is that moderated by the mechanisms of inhibition of interference?
**RQ3**: How do groups differ in maintenance of learning, and how is that moderated by the mechanisms of sustained attention?

By adopting a stage-specific neurocognitive framework, this study advances understanding of how ADHD affects learning processes, rather than merely measuring performance outcomes. Our approach aims to inform the design of adaptive interventions tailored to when and how attentional support is most beneficial for adult learners with ADHD.

## Materials and Methods

### Participants and Design

#### Participants

We recruited 53 adults (25 males, *M*_age_ = 36.26, *SD* = 10.22) diagnosed with ADHD and 18 neurotypical control adults (6 males, *M*_age_ = 32.78, *SD* = 10.82) with no diagnosed neurocognitive deficits or ongoing medication for ADHD. All participants had normal or corrected-to-normal vision. The groups did not differ in terms of age (*t*(*X*) = −0.91, *p* = .37), gender (χ^2^(1) = 0.16, *p* = .69), or handedness (χ^2^(2) = 0.48, *p* = .79). Due to reasons of data quality one participant was dropped from the ADHD sample. The control group size was 35% of the size of the ADHD group. According to statistical literature and established experimental design guidelines, while equal group sizes are ideal for maximizing power, moderate disparities do not substantially affect the validity of statistical tests, especially when robust analytical methods are used (e.g., LMM) and the total sample size is sufficiently large (Maxwell et al., 2017; Rosner, 2015; see https://www.markhw.com/blog/control-size for a simulation analysis which suggests that it is reasonable to use a control group 30% as large as the test group).

Inclusion criteria for the ADHD group were: (1) pre-existing diagnosis of ADHD, (2) no neurological diagnoses, (3) age between 18 and 60 years, (4) scores on Adult ADHD Self Report Scale (ASRS; Kessler et al., 2005) and Brown ADHD scale (BADDS; L. Davenport & Davis, 2011) indicating the presence of ADHD, and (5) an IQ score of at least 80 using WAIS IV measured by a qualified psychologist (Wechsler, 2012). No strict cut-off values were used for ASRS and BADDS to indicate the presence of ADHD. Instead, exclusion was decided by the consulting psychiatrist, who conducted structured clinical interviews with participants using the Diagnostic Interview for ADHD in Adults (DIVA 2.0; Kooij, 2017). Comorbidities were evaluated during the clinical interview, and exclusion decisions were informed by outlier scores from: Generalized Anxiety Disorder (GAD; Spitzer et al., 2006) scale, Beck Depression Inventory (BDI; Beck et al., 1996), Alcohol Use Disorders Identification Test (AUDIT; Saunders et al., 1993), the Mood Disorder Questionnaire (MDQ; Hirschfeld et al., 2000), test of prodromal symptoms of psychosis (PROD; Heinimaa et al., 2003), and the Dissociative Experiences Scale (DES; Liebowitz, 1992). The psychiatrist followed DIVA guidelines to confirm the existing ADHD diagnosis, or not.

Within the ADHD group, there was a small difference in the ASRS hyperactivity-impulsivity scores depending on the sub-type diagnosis (*F*(1,50) = 5.01, *p* < .030, *r*^2^ = .09). As expected, participants with *hyperactive-impulsive* (ADHD-HI) or *combined* (ADHD-C) diagnoses had higher hyperactivity-impulsivity scores (*M* = 6.33, *SD* = 2.56; the groups were combined) than those with an *inattention* (ADHD-I) diagnosis (i.e., without hyperactivity-impulsivity; *M* = 4.22, *SD* = 2.59). There was no significant difference in the ASRS inattention scores between groups, and all analyses treat all ADHD participants as one group.

All patients were fully briefed about all study components, were required to give informed consent for participation, and had access to a qualified psychiatrist. The study protocol followed guidelines of the Declaration of Helsinki for participants’ rights and study procedures. Approval was granted by the Ethical Committee of the Hospital District of Helsinki and Uusimaa, 28/03/2012, 621/1999, 24 \S.

#### Procedure

The behavioral and EEG data were measured in a 2 to 3 hr multi-task session conducted in an electrically-shielded and sound-attenuated room. PSICAT was administered toward the end of the measurement session. The full session included preparation (30–40 min), pre-test baseline measurement (5 min), TOVA (22 min; detailed in Cowley et al. (2022), resting state vigilance measurement (20 min), PSICAT (22 min), and a post-test baseline measurement (2 min).

Participants were asked to abstain from taking their ADHD medication for 48 hr prior to the EEG measurement (washout period). They were also advised not to take any other stimulants immediately prior to the measurement (e.g., coffee, cigarettes, and energy drinks) and to arrive as well rested as possible to the measurement. Karolinska sleepiness scale (KSS; Akerstedt and Gillberg, 1990) was employed to control for participants’ sleepiness levels, as sleepiness can affect both sustained attention and EEG measurement. Participants tended to report being alert (on the scale 1–9, ADHD: *M* = 4.60, *SD* = 1.38, controls: *M* = 3.73, *SD* = 1.39; these mean scores indicated 3 “Alert,” to 4 “Rather Alert,” and 5 “Neither alert nor sleepy”).

#### PSICAT Protocol

**Figure 1.**
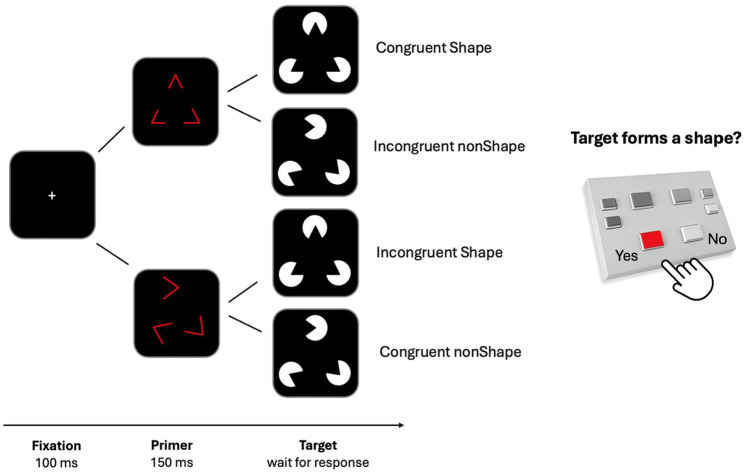
Schematic of the stimuli and protocol structure used in PSICAT. Each trial includes a fixation (100 ms), a red-line primer (150 ms), and a Kanizsa subjective contour illusion target. Four conditions are defined by primer congruency and target type. Participants respond Yes/No to whether a shape is perceived in the target, while primers are task-irrelevant.

PSICAT protocol retains the standard repetitive classification—task structure and adds complex target stimuli preceded by either Congruent or Incongruent interference primers. Primers are attention-grabbing but irrelevant to the task, and in a strategic sense should be ignored, because only target-recognition determines performance. As targets, PSICAT uses the Kanizsa subjective contour illusion (Shape; see Figure 1), which is a perceived polygon induced by collinear “Pac-Man” shapes at the vertices. When the Pac-Man shapes are not collinear, the stimulus forms a nonShape target. The task presents irregular polygons with three or four vertices, randomly located within certain constraints.

PSICAT is a two-alternative forced-choice task, where participants were asked to give fast but accurate responses with left or right hand button-press to the presence or absence of a shape in the target Kanizsa stimuli. The classification required by the task involves discrimination of gestalt images from images with identical visual features (brightness, hue, and visual angle) but no gestalt property (with an offset of the angle originating at the vertices). Congruency between the primer and the target creates a task-irrelevant probe condition. Thus, PSICAT protocol consists of 2 × 2 conditions: Congruent Shape, Incongruent Shape, Congruent nonShape, and Incongruent nonShape. The protocol begins with an explanation and set of 12 practice trials, three for each condition, to brief participants on the task under supervision of an experimenter. PSICAT consists of five blocks, each containing 110 trials and lasting approximately 5 min, with a 1-min rest period between blocks.

Example trials are shown in Figure 1. Each trial consists of a preparatory fixation cross, primer-target pair, and inter-trial interval (ITI). Fixation lasts 100 ms; primers are flashed for 150 ms; targets are held until the participant responds (750 ms on average in this study); and ITI is 500 ± 100 ms (varied to minimize presentation expectancy which reduces trial effects). Thus, an average trial should last 1.5 sec, giving an estimated protocol duration of 22 min. PSICAT protocol was designed by Cowley (2018) and is available as an open-source code repository, allowing researchers to reuse and adapt it to their requirements.

For all analyses below, both error trials and premature trials were excluded, in order to mitigate the influence of anomalous data points. Premature trials were defined using the Empirical Cumulative Distribution Function (ECDF) to calculate the fifth quantile of the RT data.

### Analysis

To examine how ADHD influences distinct stages of learning and illuminates the underlying cognitive mechanisms, we conducted a multimodal investigation combining behavioral and neural analyses (Table 1). Behavioral indices—including response time (RT) and response time variability (RTV)—are widely used to quantify overt performance in ADHD research (Gmehlin et al., 2014; Kofler et al., 2013; Leth-Steensen et al., 2000). Complementing these measures, electrophysiological indices, particularly event-related potentials (ERPs), provide millisecond-level temporal resolution, enabling precise tracking of dynamic cognitive processes such as saliency detection and interference control (Arpaia et al., 2022; Müller et al., 2020). This integrative approach offers a more comprehensive account of ADHD-related learning dynamics across the acquisition, consolidation, and maintenance phases.

**Table 1.** Cognitive Mechanisms and Analytical Measures Across Learning Stages.

Learning stage	Cognitive mechanism of interest	Block(s)	Behavioral analytic measures	Neural analytic measures	Purpose
Acquisition	Saliency processing	1	RT, RTV, Ex-Gaussian parameters	Early Occipital ERPs	Assess initial visual processing of salient stimuli
Consolidation	Interference inhibition	2–5		Parietal N2 ERP, ERSP (theta, alpha)	Evaluate ability to suppress conflicting primers and stabilize performance
Maintenance	Sustained attention	2–5		Frontal P3 ERP, ERSP (alpha, theta)	Assess capacity to maintain performance and attentional control over repeated trials

#### Neural Analysis

##### EEG Measurement and Preprocessing

EEG was measured using Biosemi ActiveTwo equipment with 128 active electrodes mounted on a cloth headcap with equiradial positions. Electrode positions are labeled in this text according to the closest International 10-5 electrode placement system label (Jurcak et al., 2007). Active electrode CMS (Common Mode Sense) and passive electrode DRL (Driven Right Leg) were used to create a feedback loop for amplifier reference. Electro-oculography (EOG) was recorded using bipolar montage: horizontal EOG electrodes were attached to the outer canthi of both eyes; vertical EOG electrodes were attached above and below the left eye. Electrode offsets (running average of voltage at each electrode) were kept below ±25 mV.

The data was preprocessed using Computational Testing Automated Preprocessing toolbox (CTAP; Cowley et al., 2017; Cowley & Korpela, 2018, based on EEGLAB (Delorme & Makeig, 2004) for MATLAB. The data was re-referenced offline to the average of the two mastoids. The data was then low-pass filtered at 45 Hz and high-pass filtered at 0.5 Hz. Each participant’s continuous EEG and EOG data was decomposed into Independent Components (ICs) using the FastICA algorithm (Hyvarinen, 1999). EEG data was cleaned from artifacts by the following automated methods, results of which were validated for each participant by visual inspection of IC activations, scalp maps, power spectrum, and ERP image.

Bad channels were detected if their variance was greater than ±3 median absolute deviations. Data for bad channels was spherically interpolated from adjacent electrodes. Eye blinks were then removed using the two-step method detailed in Cowley et al. (2017): first, blinks were detected by their outlying time domain derivative, and labeled; second, ICs statistically similar to the detected blinks were removed (Cowley et al., 2017). Fluctuations exceeding ±150 µV in amplitude across >15% of channels were discarded as bad segments. Next, ICs representing artefacts other than blinks were detected using FASTER (Fully Automated Statistical Thresholding for EEG artefact Rejection; Nolan et al., 2010) toolbox.

All neural analyses focused on three ROIs: frontal at FC2, F2, F4h, AFFz, Fz, FC1, F1, F3h, FCC4h, and FCC3h, parietal at CPPz, P1, CPP3h, Pz, PPOz, P2, and CPP4h, and occipital at I1, OI1, O1, POO3, POOz, Oz, OIz, Iz, I2, OI2, O2, and POO4. This selection of electrodes ensures a broad bilateral coverage of the corresponding lobes, which are known to play a significant role in visual attentional processes and response inhibition (Coull, 1998; Di Russo et al., 2002; Folstein & Van Petten, 2008; Hanslmayr et al., 2005; Luck, 2005).

##### Event-related Potentials Calculation

Epochs were generated from the EEG data using MATLAB, time-locked to the target onset of hit trials only (excluding error trials). Continuous EEG was split into 750 ms epochs: −250 to −150 ms baseline, −150 to 0 ms primer, and 500 ms after target stimulus onset. Epochs were baseline-corrected with respect to the mean voltage of the 100 ms period preceding the primer onset. After baseline-correction, a 20 Hz low-pass filter was applied for visualization and testing.

We conducted ERP analyses by averaging EEG signals across trials and participants for each condition and block, producing group-level ERP waveforms that reflect the typical neural response over time. To statistically assess differences between groups, we used the minimum-width envelopes (MWEs) method (Korpela et al., 2014). MWE approach constructs non-parametric confidence bands around the group mean waveforms. Statistically significant differences are identified by examining time intervals where these confidence bands do not overlap, indicating distinct temporal patterns in ERP morphology between groups. The set of topographic scalp maps were built to represent the distribution of corresponding electrical potentials across the ROIs. Time ranges were selected based on morphology of MWE waves in the corresponding area.

To examine MWE ERPs for RQ1, we focused on occipital ROI in Block 1 as this brain region most reflecting early processing of salience (Corbetta & Shulman, 2002) and this time period corresponds to the stage of learning acquisition (see RQ1: Acquisition Phase Section for more details). To track the changes in neural processing over time (RQ2 and RQ3), we compared MWEs and corresponding topographic scalp maps between ADHD and Control groups in Blocks 2 to 5, following the acquisition phase, at parietal and frontal regions of interest. Parietal ROI, particularly the posterior parietal areas, plays a particularly important role in in attentional control and filtering out irrelevant information (Corbetta & Shulman, 2002; Culham & Kanwisher, 2001). At the same time, neuroimaging studies have shown that frontal activation is strongly correlated with sustained attention, particularly in tasks that require individuals to avoid lapses in concentration (e.g., continuous performance tasks; Callicott et al., 2003; Posner & Petersen, 1990).

##### Event-related Spectral Perturbation (ERSP) Calculation

To examine event-related oscillatory power dynamics for correct trials, ERSPs (Makeig et al., 2004) were calculated within the frontal, parietal, and occipital ROIs defined above. We sampled 15 out of 27 trials per participant, condition, and block; merged them within groups, and finally sampled as many trials from the larger ADHD group as was included in the smaller Control group. The number of trials were thus balanced to ensure that any observed effect would be due to altered neural processing, not merely due to possible differences in behavioral performance (e.g., amount of correct trials). Spectral power was calculated in 54 log-spaced frequencies from 3 to 30 Hz, in 200 time points (−187 to 394 ms), using Morlet wavelets with cycles increasing linearly from 1.1 to 11 in windows of 209 samples (408 ms). Figures display the average ERSP matrices from all ROI electrodes.

The group-wise difference of base 10 log power of the ERSPs was tested for statistical significance using permutation testing based on EEGLAB function “condstat.” Significance was computed at two levels, α = .05 (200 permutations) and α = .0005 (2,000 permutations), to illustrate a robust test statistic.

#### Behavioral Analyses

Statistical analyses of behavior were conducted in R, with linear mixed-effects models (LMMs) used for handling trial-level and block-level RT data. LMMs were chosen for their ability to accommodate nested data structures, correlated observations, and unbalanced designs (Gelman & Hill, 2006). Post hoc comparisons were conducted using generalized linear hypothesis tests (glht, *multcomp* package) and joint significance tests (*emmeans* package). Additionally, we quantified standardized effect sizes using Cohen’s *d*, computed from model-estimated contrasts and pooled standard deviations. Family-wise error rates were controlled using the Holm–Bonferroni adjustment.

To manage extreme responses, we excluded the lowest 0.05% of RTs to remove potential anticipatory responses or measurement errors. Points in the upper tail of the RT distribution, representing slower responses, were captured by the tau (τ) parameter of the ex-Gaussian distribution, and thus not excluded. The ex-Gaussian model consists of two components: the Gaussian component, which captures the mean RT (μ) and the standard deviation (σ), and the exponential component, which accounts for the tail (τ) of the distribution, reflecting abnormally slow responses often attributed to attentional lapses or momentary disengagement. This approach is particularly well-suited to the right-skewed and highly variable nature of RT data commonly observed in ADHD populations. By decomposing RT into these components, the ex-Gaussian distribution allows for a more detailed analysis of both the central tendency and the slower, more variable responses in ADHD, which are crucial for understanding the attention-related delays and response time variability (RTV) in this population (Burbeck & Luce, 1982; Dawson, 1988; Lee et al., 1998).

##### Task Protocol Validation: Congruency and Shape Effects

We first provide evidence that PSICAT reliably engages key cognitive mechanisms, isolates the impact of the primer-target relationship and predicts behavioral performance. Once established, this paradigm serves as the foundation for the study’s RQs. To validate task sensitivity, we tested the effects of Congruency (Congruent vs. Incongruent) and Shape (Shape vs. nonShape) conditions on RTs. A robust LMM was fitted with log-transformed RT as the dependent variable, and Group, Congruency, Shape, and their interactions as fixed effects. Random intercepts were included for Subject, Congruency × Subject, and Shape × Subject to allow individual baseline variation. Ex-Gaussian parameters were also modeled in a follow-up LMM to examine how Congruency and Shape impacted the shape of the RT distributions across groups. Random intercepts were specified for Subject and interaction terms, tau was log-transformed.

##### Main Analyses: Learning Acquisition, Consolidation, and Maintenance

To investigate how do ADHDs and Controls differ in learning acquisition (RQ1), we first established where this most active phase of learning occurs. We applied a subject-wise rolling window mean on RTs across trials and defined the end of active learning as the first instance where three consecutive windows fall within 1.5 *SD* of the mean from the ex-Gaussian distribution fitted to all RTs, which captures the “core” RT process, excluding outliers and the long tail). Figure 3 illustrates the progression of RTs across blocks, highlighting a rapid decline within the first block, followed by gradual stabilization in subsequent blocks. After the cutoff point was identified, an LMM was fitted with log-RT as DV, and log-Trial, Group, and their interaction as fixed effects, with random intercepts and slopes for Trial.

RT variability (RTV) was assessed via rolling window variance across trials, then converted into *SD*s. Log-transformed *SD* served as DV, with group (ADHD, Control), window (cubic-transformed to account for its non-linear relationship with *SD*), and their interaction as predictors. A random intercept captured subject-level baseline differences.

To address RQ2 and RQ3 (consolidation and maintenance), we modeled RT and variability (*SD*) across Blocks 2 to 5, using block-wise trials (bwTrial) or rolling windows (bwWindow), respectively. RT and *SD* variables with skewed distribution were log-transformed. LMMs included fixed effects for Group, Block, and bwTrial (or bwWindow), along with all two-way and three-way interactions. Block was treated as an ordered factor with separate intercepts. Random intercepts for block–subject and trial–subject combinations allowed baseline variation.

For all RQs 1 to 3 we advanced our analysis by calculating Subject-level ex-Gaussian parameters for RT data and fitted them in a LMM, with mu, sigma and tau as the DV, and group (ADHD, Control), Block (when applicable) and their interaction, as the predictors. Tau variable had skewed distributions so was log-transformed. Subjects’ ID was used as a random factor. When appropriate, random slopes for the Block-within-Subject were added to model variation in learning trajectories.

Table 1 outlines how each learning phase is linked to distinct cognitive mechanisms (saliency processing, interference inhibition, and sustained attention), with associated behavioral and neural measures. The purpose of these analyses is to assess key cognitive functions at different stages of learning, from initial processing of salient stimuli in the acquisition phase, to the ability to stabilize performance and manage attentional control over time.

## Results

**Figure 2.**
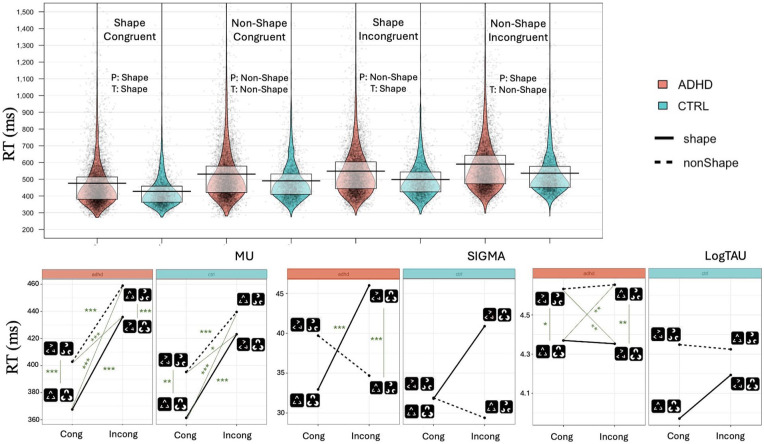
Top: Violin plots of RTs for hit trials in each individual condition. Black line represents the mean and the gray box—first and third quartiles. Bottom: Ex-Gaussian parameters (mu, sigma and tau) for individual conditions.

To verify that PSICAT protocol is functioning as intended, we use an LMM with RT as DV to investigate the effect of Shape and Congruency conditions. There was no main effect of Group (marginal), but there was a prominent effect of Congruency on RTs (β = .13, 95% CI [0.12, 0.14], *p*_adj_ < .001) which corresponds to an approximate 13.7% increase (on original RT scale) in RTs for Incongruent trials relative to Congruent trials. Similar effect was also found for the effect of Shape on RTs (β = .09, 95% CI [0.07, 0.11], *p*_adj_ < .001) with the Shape condition being approximately 9.5% faster than nonShape. Condition RTs were ordered as shown in Figure 2. RTs increased first by Congruency and then by Shape for both groups, such that *Congruent shape* condition was the fastest among all other conditions and *Incongruent nonShape* was the slowest.

Digging deeper into ex-Gaussian parameters of trial-wise RTs, we did not find any statistically significant difference between the groups in mu parameter; its patterns showed clear similarities for both groups (Figure 2). There was large significant main effect of Congruency on mu (β = 57.6, t(128)=8.38, *p*_adj_ < .001) with a more pronounced effect on ADHD group RT than on Controls’ (β = −12.60, 95% CI [−24.64, −0.55], *p* < .05). Thus, the increase in mu from Congruent to Incongruent trials is 12.60 ms greater in the ADHD group compared to the Control group. We also found significant effect of Shape (*t*(105) = 3.6, *p*_adj_ < .001) with a medium-size (β = 27.2, 95% CI [17.6, 36.7], *p*_adj_ < .001) with no difference between the effects on both groups.

Groups were not different in sigma parameter of ex-Gaussian distribution. The post-hoc analysis (Figure 2) revealed that ADHD group sigma parameter was larger in response to *Incongruent Shape* condition in comparison with both *Congruent Shape* (*t* = −5.58, *p*_adj_ < .001) and *Incongruent nonShape* (*t* = 11.32, *p*_adj_ < .001) conditions, while for Control group there was no difference between these conditions.

For tau, main effect of Group was large (β = .29, *t*(132) = 2.35, *p* < .05, *p*_adj_ = ns). On the original scale, this corresponds to a 34% increase in tau in the ADHD group compared to the Controls. Looking at each condition separately, we found statistically significant difference between Shape and nonShape targets on log-transformed tau regardless of the Congruency for ADHD group (*t* = −3.40, *p*_adj_ < .05 for Congruent conditions, *t* = −3.89, *p*_adj_ < .01 for Incongruent conditions), but not for Controls.

To gain a deeper understanding of how early visual processing stages influence subsequent target processing and to establish direct neural-behavioral correlations, we conducted ERP analyses for each individual condition (included in the Supplemental Materials for being out of scope of the study of learning).

### RQ1: Acquisition Phase

**Figure 3.**
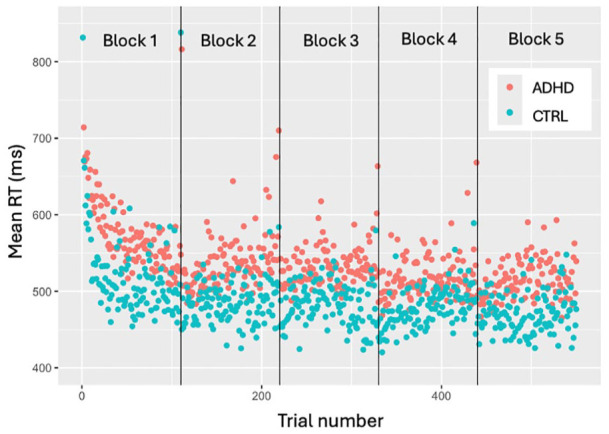
Group-wise mean RTs across trials.

To study the differences between ADHD and Control groups in initial learning stage we first identified the cutoff point (where three consecutive data points fall within 1.5 *SD* of the mean of ex-Gaussian distribution). It was established at trial 112 out of 550, which nearly coincides with the end of Block 1 (Trial 110). Our results were supported visually, demonstrating a substantial decrease in mean RTs in the first Block of experiment, followed by relatively stable performance across subsequent blocks (with some increase in RTV within each block; see Figure 3). We attribute this decline to participants’ initial acquisition of skill to perform. In this time-period of interest (Block 1), LMM model revealed no main effect of Group after adjustment for multiple comparisons. We found main effect of log-transformed Trial (β = −.08, *t*(68) = −10.19, 95% CI [−0.10, −0.07], *p*_adj_ < .001), while Trial-Group interaction also did not demonstrate significant effect on RT.

Variability analysis revealed the main effect of Group (β = −.18, *t*(6,077) = −13.48, 95% CI [−0.21, −0.16], *p*_adj_ < .001) on *SD*, implying 16.6% reduction in *SD* (on original *SD* scale) in the Control group compared to the ADHD group. Ex-Gaussian analysis of RT variables demonstrated no main effect of Group on mu and sigma parameters. According to Welch Two Sample t-test, Group variable has a marginally significant effect (*t*(28) = 1.87, *p* = .07, *p*_adj_ = ns, Cohen’s *d* = 0.45) on tau.

**Figure 4.**
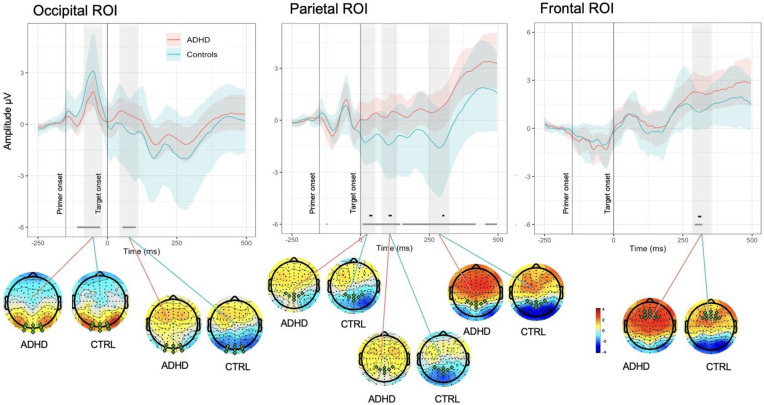
Spatial and temporal activations shown by grand average ERPs (calculated for ROIs) and associated whole-head scalp maps (calculated for the time window of curve testing). Each plot shows the average ERPs responses (solid lines) of the ADHD (red) and Control (blue) groups. The mean responses are surrounded by the MWE confidence bands of the time series. The significance lines at the bottom of each plot denote time points where group ERPs were drawn from different distributions (gray: mean of one group is outside MWE of the other group; black: mean of both groups are outside each other’s MWE). Note the negative deflection peaking around 300 ms in Frontal ROI in Control group, while weaker and delayed in latency peak in ADHDs, creating significant differences; in Parietal ROI reduced amplitude of N2 component demonstrated by ADHD group in comparison with Controls.

**Figure 5.**
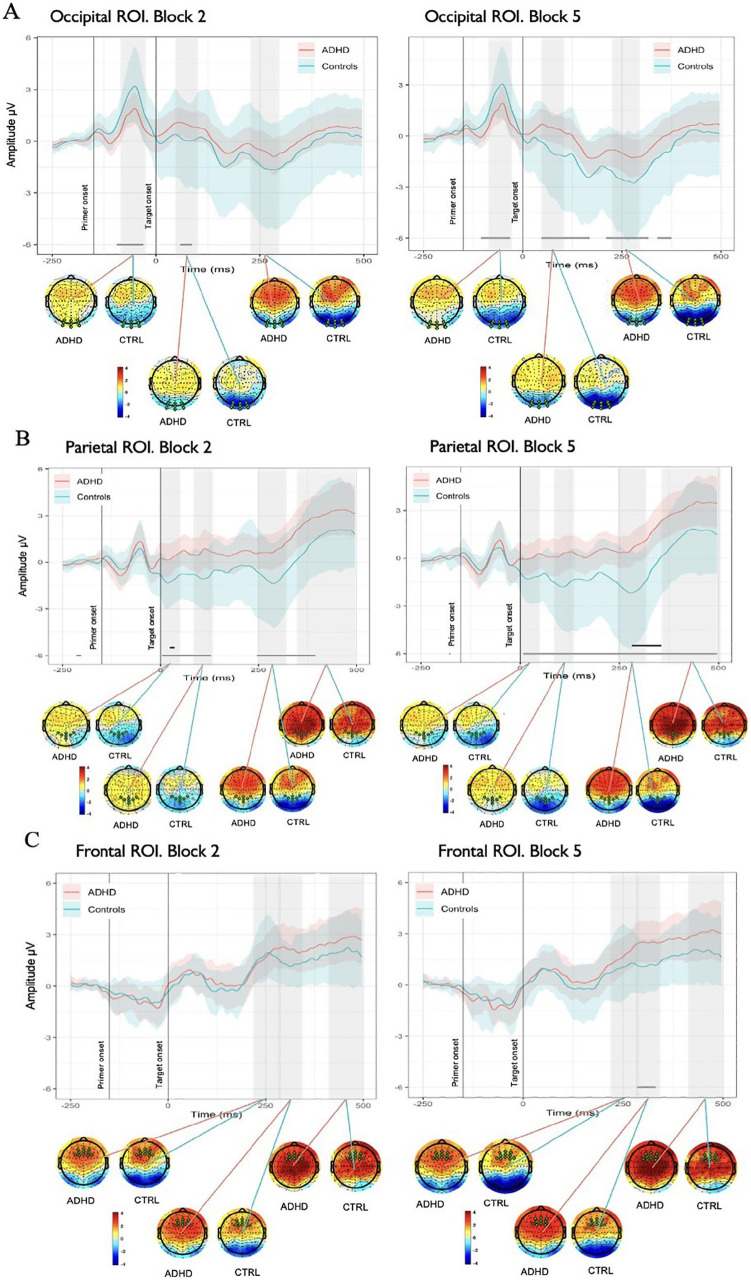
(A) Spatial and temporal activations shown by grand average ERPs in Occipital ROI in Block 2 versus Block 5 and associated whole-head scalp maps calculated for the time window of curve testing. Each plot shows the average ERPs responses (solid lines) of the ADHD (red) and Control (blue) groups. The mean responses are surrounded by the MWE confidence bands of the time series. The gray significance lines at the bottom of each plot denote time points where group ERPs were drawn from different distributions. (B) Spatial and temporal activations shown by grand average ERPs in Parietal ROI in Block 2 versus Block 5 and associated whole-head scalp maps. Black significance lines indicate time points where differences met a stricter significance criterion. (C) Spatial and temporal activations shown by grand average ERPs in Frontal ROI in Block 2 versus Block 5 and associated whole-head scalp maps.

Analysis of MWEs in the occipital area, revealed significantly reduced activity in reaction to primer in ADHD group in comparison with Controls 50 to 120 ms after the onset of primer (see Figure 4). Later on, de-synchronization demonstrated by the Control group around 200 to 250 ms after the primer onset (50–100 ms after the target onset) drove significant differences between ADHD and Controls in the corresponding time period, giving insights on distinct saliency processing mechanisms employed by two groups in the acquisition phase of learning. Comparison across the time of experiment revealed differences between the groups across longer periods of epoch in Block 5 in comparison to Block 2 (see Figure 5(A)).

### RQ2 and RQ3: Consolidation and Maintenance Phases

**Figure 6.**
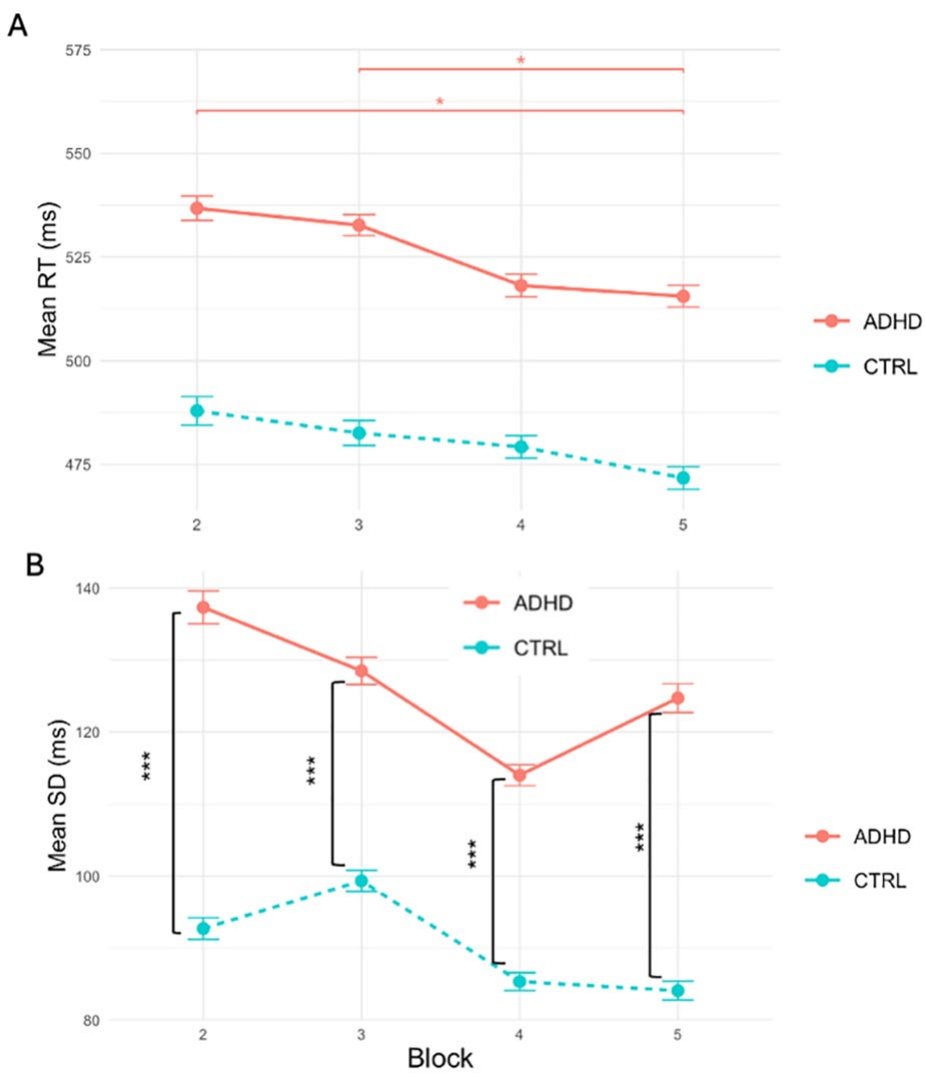
(A) Mean RTs across Blocks for ADHD and Control groups. Error bars represent the standard error of the mean. Significant differences are indicated by asterisks: *p < .05. Narrow error bars signify the good model fit. Despite the model’s accuracy, the actual differences in RTs between the groups are negligible. (B) Mean RTVs across Blocks for ADHD and Control groups. Error bars represent the standard error of the mean. Significant differences are indicated by asterisks: ***p < .001. Groups are different in RTV across all Blocks.

Studying the question of learning consolidation and maintenance we focused our analysis on the Blocks following the acquisition phase. We built similar LMM model and added the Block variable to track the participants’ performance over time. Our model revealed no main effect of Group or Block on RT. Post-hoc analysis demonstrated that for ADHD group RTs in Block 2 and 3 were significantly higher than in Block 5 (β = .04, *z* = 3.43, 95% CI [0.02, 0.06], *p*_adj_ < .05, and β = .03, *z* = 3.13, 95% CI [0.01, 0.05], *p*_adj_ < .05, respectively). For Control group, no differences were found in performance between blocks, highlighting the differences in learning consolidation between the two groups (see Figure 6(A)).

**Figure 7.**
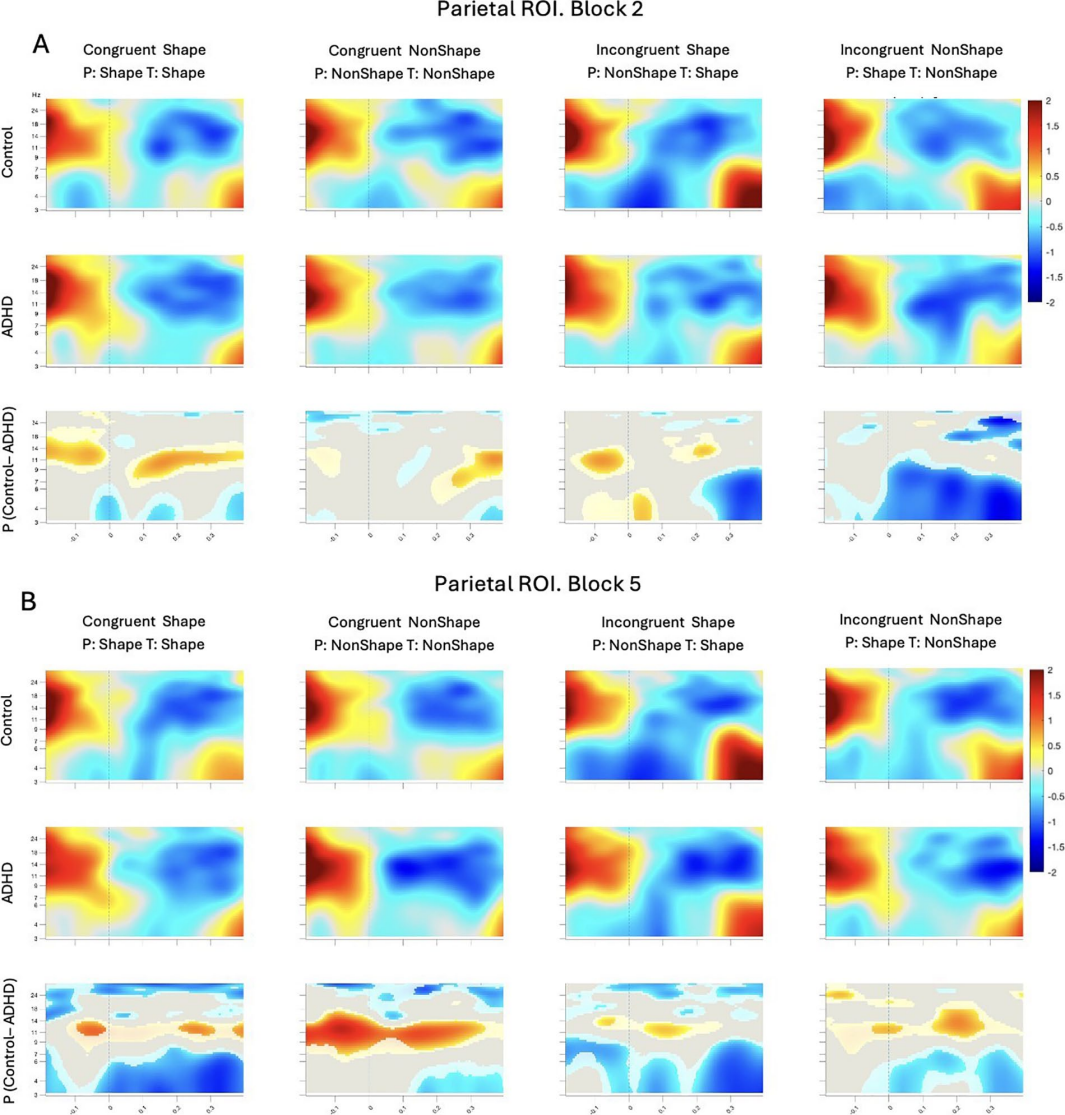
ERSP plots for correct trials within parietal ROI for Block 2 (A) and Block 5 (B). ERSPs are locked to target onset at time 0 (dashed lines). Top and middle rows show ERS/ERD for Control and ADHD groups, respectively. The time-frequency data is averaged across all electrodes in the ROI. The bottom row shows the mean difference between groups for the ROI-averaged time-frequency data; these plots are masked by a permutation-based significance test: gray is n.s., lighter-toned blobs are different at *p* < .05, and full-color blobs are different at *p* < .0005 (all tests uncorrected).

Our analysis of RTV revealed the main effect of Group (*t*(15,510) = −6.34, *p*_adj_ < .001) with a substantial magnitude (β = −.23, 95% CI [−0.25, −0.22], *p*_adj_ < .001), translating to a 20.6% reduction in *SD* in the Control group compared to the ADHD group. Both groups showed decreased *SD*s throughout the experiment, although the dynamics were not monotonic or significant. Joint tests and post-hoc analysis revealed significant differences between the groups in *SD* in all Blocks (Block 2: *F*(1, 13,529) = 235.07, *p*_adj_ < .001; Block 3: *F*(1, 13,535) = 119.30, *p*_adj_ < .001; Block 4: *F*(1, 14,756) = 23.44, *p*_adj_ < .001; Block 5: *F*(1, 14,670) = 49.97, *p*_adj_ < .001, see Figure 7(B)). Ex-Gaussian analysis of RTs demonstrated no main effect of Group on mu and sigma. We found a 22.1% reduction in tau for the Control in comparison with ADHD group, which was nevertheless lacking statistical significance.

In parietal ROI, differences between groups were present intermittently throughout the length of the epoch, with the most prominent ones corresponding to negative peaks in around 20, 110, and 300 ms demonstrated by Control group (see Figure 4). ADHD group had a similar morphology of the MWE wave but with delayed latency of peaks and weaker activation, especially for the last negative peak around 300 ms after target onset. Similar to the other ROIs, in parietal area differences between the groups were present across longer periods of epoch in Block 5 in comparison to Block 2 and the effects (positivity or negativity) intensified toward the end of experiment (see Figure 5(B)).

To complement these ERP findings and further examine group-specific neural dynamics, we conducted a condition-wise ERSP analysis in the parietal region. Figure 7 shows parietal baseline-corrected ERSPs for Block 2 in panel A and Block 5 in panel B. ADHD group demonstrated stronger activity than Controls in alpha (9–ƒ14 Hz) frequency band consistently across the epoch. The differences became stronger by Block 5, suggesting overall increase of alpha power in ADHD. Control group demonstrated stronger temporary locked theta ERS (3–8 Hz) than ADHD in Incongruent conditions in Block 2, which grew in strength by Block 5 and became the strongest in *Congruent Shape* condition, following by *Incongruent Shape*. Notably, overall ERSP differences between the groups became stronger in Block 5 in comparison with Block 2. Other areas of difference did not show such consistent temporal patterns and are thus not considered further.

Further focusing on differences in maintenance of performance and sustaining focus over prolonged periods of time, our analysis of frontal ROI demonstrated that Controls had a negative deflection peaking around 300 ms after target onset (see Figure 4), while weaker and delayed in latency peak is noticeable in the ADHD group, driving the significant differences between the groups. ADHD group demonstrated overall enhanced positive activity in comparison to Controls from 250 ms after the target onset. The comparison across blocks revealed differences between ADHDs and Controls in Block 5 and absence of such in Block 2 (see Figure 5(C)). Topographic maps showed stronger positive activation within ADHD group rather than in Control in 220 to 290, 290 to 350, and 400 to 500 ms time ranges, though more prominent centrally in the latter one. Both groups slightly increased intensity of positive activation from Block 2 to Block 5 in two latest time ranges of interest.

## Discussion

The current study investigated how the cognitive mechanisms of saliency processing, inhibition of interference, and sustained attention differ between adults with ADHD and neurotypical Controls, during the acquisition, consolidation, and maintenance phases of learning. Our results revealed significant differences in neural and behavioral responses between ADHD and Control groups, highlighting distinct cognitive processes and compensation mechanisms in individuals with ADHD.

As a first step, we verified the PSICAT protocol validity. PSICAT effectively isolated the impact of the primer-target relationship, with Congruency showing a stronger influence on reaction times than Shape. This suggests that the task-irrelevant relationship between the primer and target was more influential on task performance than the task-relevant target alone, in accordance with Cowley (2018). Additionally, both Congruency and Shape produced an effect on mu parameter of ex-Gaussian distribution in both groups, highlighting the importance of both features in shaping main response patterns. Notably, nonShape targets led to response slowing in both ADHD and Control groups, reflected in increased tau values. This aligns with the understanding that tau is by definition the late response occurring after full target processing—by which point the primer’s influence may have diminished. Overall, these results confirm that the PSICAT protocol effectively engaged the targeted cognitive mechanisms of saliency processing and interference inhibition in both ADHD and Control participants.

In the next sections, we address the research questions related to different stages of learning and the underlying cognitive mechanisms.

### Acquisition Phase

In our experiment, learning to perform the task implied judging the implicit contours of target shapes, whose visual saliency was modulated by congruency of the preceding primer. The occipital lobe plays a crucial role in processing visual information and illusory contours, in particular (Corbetta & Shulman, 2002). According to the previous literature (De Valois et al., 1982; Lee et al., 1998; Pollen, 1989), approximately 40 ms after stimulus onset individual V1 neurons in occipital area recognize simplest object features and can discriminate small changes in visual orientations, spatial frequencies, and colors. Smaller amplitudes in ADHD group in response to angled line stimuli around 65 to 125 ms after primer onset in our experiment (Figure 6(A)) might represent impaired bottom-up mechanisms in visual processing in adults with ADHD. This aligns with previous studies that have highlighted alterations in bottom-up attention processes in ADHD patients (Janssen et al., 2016; Schneidt et al., 2018; Sergeant et al., 2002).

While visual detection of object is an automatic process, full perceptual completion of gestalt stimuli implies awareness and feedback from higher visual areas back to lower ones. First, stimuli are processed automatically in V1/V2, then sent forward to lateral occipital complex to synthesize illusory contours, segment it and assign boundaries, and later sent back to V1/V2 to fill in details, complete the figure percept, and integrate contextual information (Koivisto et al., 2011; Rao & Ballard, 1999). This two-stage model is supported by studies suggesting that full perceptual completion of illusory contours occurs approximately 160 ms after stimulus onset (Keane et al., 2016; Marini & Marzi, 2016; Wokke et al., 2013). In our experiment, differences between ADHD and Controls found 200 to 250 ms after primer onset in occipital ROI might represent the later integration stage of visual processing and reveal differential engagement of top-down visual processing mechanisms in individuals with ADHD.

Neural alterations in visual processing find partial confirmation in our behavioral results, demonstrating less consistent responses in ADHD group in comparison with Controls in Block 1. The RTV was particularly larger when processing trials with nonShape primers, presumably corresponding to greater uncertainty of perceptual processing, requiring more cognitive resources. Notably, ADHDs and Controls were also different in the number of attentional lapses, reflected in tau component of ex-Gaussian distribution. Presumably, slightly larger number of abnormally slow responses (tau) within the ADHD group drives the differences in RTV between ADHDs and Controls, which is in line with background literature (Epstein et al., 2010; Kofler et al., 2013; Leth-Steensen et al., 2000; Russell et al., 2006; Tarantino et al., 2013). For instance, Leth-Steensen et al. (2000) demonstrated that increased RTV in a simple reaction time task in participants with ADHD was primarily associated with heightened tau parameter, with no significant differences in mu and sigma compared to typically developing controls. It is thus crucial to stress the importance of ex-Gaussian distribution in comparison with conventional measures of central tendency. Finally, we found no differences between the groups in speed of performance for both RT and Ex-Gaussian distribution analyses.

Overall, our findings suggest that while individuals with ADHD can perform the task at a speed comparable to Controls, they show reduced neural activation during the initial stages of visual processing. This reduced activation likely contributes to their less consistent performance, which is rooted in a higher number of attentional lapses. In essence, while ADHD individuals demonstrate a capacity to learn the task, disruptions in processing salient visual cues may undermine the stability of learning, particularly in early stages when new rules or distinctions are being internalized.

### Consolidation Phase

After individuals acquire the ability to perform a task, the next critical step is consolidating that learning—stabilizing performance and building resistance to interference. In ADHD, this phase tends to be delayed or prolonged (Ballan et al., 2022). One contributing factor is the difficulty individuals with ADHD face in managing distractions that can significantly affect how well information is consolidated and later recalled (Dvorak, 2024).

Our ERP findings support this distinction. In our experiment the largest amplitude negative peak, according to its location and latency, corresponded to the N2 component of ERPs, which reflects response inhibition, conflict detection, and error monitoring (Folstein & Van Petten, 2008; Patel & Azzam, 2005). N2 amplitudes were significantly more prominent in Incongruent trials, consistent with the mismatch between primed expectation and the actual shape of the target. Importantly, while Control group demonstrated strong negative deflection in the time range of interest, for ADHD group the N2 component was attenuated, creating statistical differences between the groups. This reduction in conflict-related activity aligns with findings of diminished N2 responses in adult ADHD during cognitive control tasks (McLoughlin et al., 2009; Woltering et al., 2013), suggesting impaired interference resolution during consolidation.

Beyond ERPs, time-frequency analyses revealed additional group differences in oscillatory activity. The amount of ERS in the theta range was higher for the Control group in comparison with ADHD for Incongruent trials in Block 2. The differences between the groups in the theta band became even stronger in Block 5. These findings mirror those of Nejati and Ghayerin (2024), who found significantly less theta rhythm and event-related desynchronization during inhibition and response trials in visual CPT.

Behavioral results further reflect these neural inefficiencies. Across Blocks 2 to 5, the ADHD group responded on average 50 ms slower than the Control group, though not significantly so. However, while Control group RT plateaued early, ADHD group continued to improve, with significantly faster responses in Block 5 compared to Blocks 2 and 3. This pattern suggests an extended consolidation phase for ADHD participants: although slower to stabilize performance, they gradually catch up, consistent with the idea that consolidation in ADHD is delayed but not impaired (Ballan et al., 2022).

In summary, our findings indicate that learning consolidation in ADHD is characterized by slower progression and increased neural inefficiency, rather than an outright failure to consolidate. Reduced N2 amplitudes and blunted theta synchronization point to a weakened ability to inhibit misleading cues and sustain top-down control, complicating the transition from skill acquisition to stable performance. Yet, the gradual improvements seen in RTs suggest that given sufficient exposure and relevant feedback, ADHD learners are capable of consolidating knowledge effectively. These insights underscore the importance of minimizing interference and designing learning environments that support sustained engagement over time for individuals with ADHD.

### Maintenance Phase

A key aspect of experiment in Blocks 2 to 5 is the repeated exposure to similar stimuli, necessitating the application of previously acquired-and-consolidated learning, over an extended period. This process demands sustained attention—continuous and self-directed awareness of a subset of stimuli, that occurs when responding to approximately uniform task stimulation (Robertson et al., 1997; Unsworth & Robison, 2020).

Behaviorally, the ADHD group in our study did not demonstrate slower mean RTs compared to Controls during Blocks 2 to 5, but did show increased RTV, consistent with previous findings (Kofler et al., 2013). Notably, ex-Gaussian analyses revealed no significant group differences in mu and sigma parameters, but showed marginal effects for tau, which reflects infrequent, long-latency responses often linked to deficits in sustained attention (Gmehlin et al., 2014). This aligns with the interpretation that while mean performance may appear intact, sustained attention is disrupted by brief, intermittent lapses in ADHD. One possible explanation for their preserved mean performance lies in the “decoupling hypothesis” (Smallwood, 2013). This theory suggests that during mind wandering, cognitive resources shift away from the primary task to internally generated thoughts. While this shift typically results in reduced performance during the wandering episode, it may also enable a resetting of cognitive resources, which can lead to better post-wandering performance on the task at hand when the mind returns to it (Smallwood, 2013).

Neurophysiological data reinforced these behavioral findings. In our study, ADHD participants exhibited reduced frontoparietal theta-band activity and elevated alpha power over time, especially toward the later blocks of the experiment. Reduced theta activity has been linked to impaired attentional sampling and deficient engagement of top-down control processes (Cowley et al., 2022). Elevated alpha activity is typically linked to fatigue or reduced attentional engagement, further supporting the interpretation that sustained task demands disproportionately burden ADHD individuals over time (Fan et al., 2015; Wascher et al., 2014).

Interestingly, although the ADHD group did not slow behaviorally, their elevated variability and neurophysiological markers suggest that maintaining task performance came at a higher cognitive cost. This was further supported by our ERP data: both groups showed comparable early frontal activity after target onset, but the ADHD group demonstrated increased late frontal P3 amplitude starting from 250 ms post-stimulus. While the P3 is typically reduced in ADHD populations (Hasler et al., 2016; Polich, 2007), in our study, the ADHD group exhibited enhanced and prolonged P3 responses relative to Controls.

The P3 component is often interpreted as reflecting processes of attention allocation, working memory updating, and decision-making (Kok, 2001; Kutas et al., 1977). Enhanced late P3 in ADHD participants may suggest compensatory recruitment of cognitive control resources to offset earlier inefficiencies in sensory or attentional processing. This interpretation is consistent with previous ERP studies reporting delayed latencies and altered topography of P3 in ADHD (Grane et al., 2016; Senderecka et al., 2012; Sokhadze et al., 2012). Notably, Sokhadze et al. (2012), using regular-shaped Kanizsa targets, also reported delayed but enhanced late ERP components in ADHD, possibly reflecting reactive rather than proactive control mechanisms.

Taken together, our findings suggest that while adults with ADHD can engage with and retain task-relevant information, their ability to maintain consistent performance over time is compromised. This deficit is not always reflected in average response times but becomes evident in variability, neural engagement, and compensatory activation patterns. Elevated alpha and reduced theta power, combined with atypical ERP dynamics, highlight that the maintenance of learning in ADHD may be achieved through greater neural effort and at the expense of sustained attention. These findings underscore the importance of considering not only task accuracy or speed, but also the stability and neurocognitive cost of performance over time in adult ADHD.

### Practical Implications and Future Research

This study provides a stage-specific analysis of learning in adults with ADHD, revealing distinct neurocognitive dynamics. Crucially, our work extends existing models of ADHD by offering a more nuanced understanding of how attentional and cognitive control mechanisms unfold over time. This temporal dissection of learning reveals that adults with ADHD are capable of effective learning but require tailored supports aligned with distinct phases of the learning process. During acquisition, reduced visual saliency processing in ADHD highlights the benefit of using perceptually clear, well-cued instructional materials (e.g., Kalanthroff et al., 2013). In the consolidation phase, where interference resolution is critical, low-distraction environments and interventions aimed at improving conflict monitoring (e.g., cognitive training or neurofeedback) may be particularly effective (Elbe et al., 2023; Osborne et al., 2023). For the maintenance phase, where sustained attention is compromised, strategies such as scheduled cognitive breaks, environmental re-engagement cues, and adaptive pacing can help mitigate attentional fatigue and support long-term task engagement (Tegelbeckers et al., 2022; Tucha et al., 2017).

Future research should further investigate the temporal dynamics of attentional engagement in ADHD using multimodal neuroimaging and real-world task designs that mimic the cognitive demands of everyday learning and work environments. Exploring individual differences within the ADHD population—such as symptom subtypes, comorbidities, or medication status—may clarify the variability in neural engagement and behavioral outcomes observed here. Intervention studies targeting phase-specific deficits, such as cognitive control training during consolidation, or neurofeedback to enhance frontal theta during maintenance, would be valuable for testing the practical implications of the current findings. Ultimately, tailoring support to distinct learning phases holds promise for more precise and effective approaches to improving functional outcomes in adults with ADHD.

## Conclusion

This study revealed distinct neural profiles in how adults with ADHD and neurotypical Controls process gestalt visual stimuli across different stages of learning during an ecologically-valid CPT. Although behavioral performance between groups was broadly comparable in terms of response speed, individuals with ADHD consistently exhibited greater response time variability and intermittent attentional lapses—hallmarks of sustained attention difficulties. Neurophysiologically, the ADHD group demonstrated reduced activation in occipital and parietal regions during early stages of visual processing, suggesting weakened bottom-up saliency detection and increased susceptibility to distraction. However, during later stages, they showed heightened frontal synchronization, particularly in the form of amplified late P3 components, which likely reflect compensatory recruitment of cognitive control mechanisms. These findings underscore that while adults with ADHD can learn and perform at levels similar to neurotypical peers, they do so via different and often more effortful neural pathways. Our results contribute to a deeper understanding of the temporal and neurocognitive dynamics of learning in ADHD and highlight the importance of adaptive, stage-sensitive support strategies. This work has practical implications for optimizing educational and occupational environments to better accommodate attentional regulation challenges in ADHD.

## Supplemental Material

sj-docx-1-jad-10.1177_10870547251356744 – Supplemental material for Mechanisms of Learning in Adults With ADHD During an Ecologically-Valid Visual Discrimination Task Supplemental material, sj-docx-1-jad-10.1177_10870547251356744 for Mechanisms of Learning in Adults With ADHD During an Ecologically-Valid Visual Discrimination Task by Elizaveta Kuznetsova, Tuisku Tammi, Natalia Postnova, Jussi Palomäki and Benjamin Ultan Cowley in Journal of Attention Disorders
