# Supplementary material for: Mechanisms of Learning in Adults With ADHD During an Ecologically-Valid Visual Discrimination Task
Source: J Atten Disord. 2025 Aug 7;30(1):38–56. doi: 10.1177/10870547251356744 (PMC12686185; doi:10.1177/10870547251356744)
Supplement: sj-docx-1-jad-10.1177_10870547251356744 – Supplemental material for Mechanisms of Learning in Adults With ADHD During an Ecologically-Valid Visual Discrimination Task [file sj-docx-1-jad-10.1177_10870547251356744.docx]

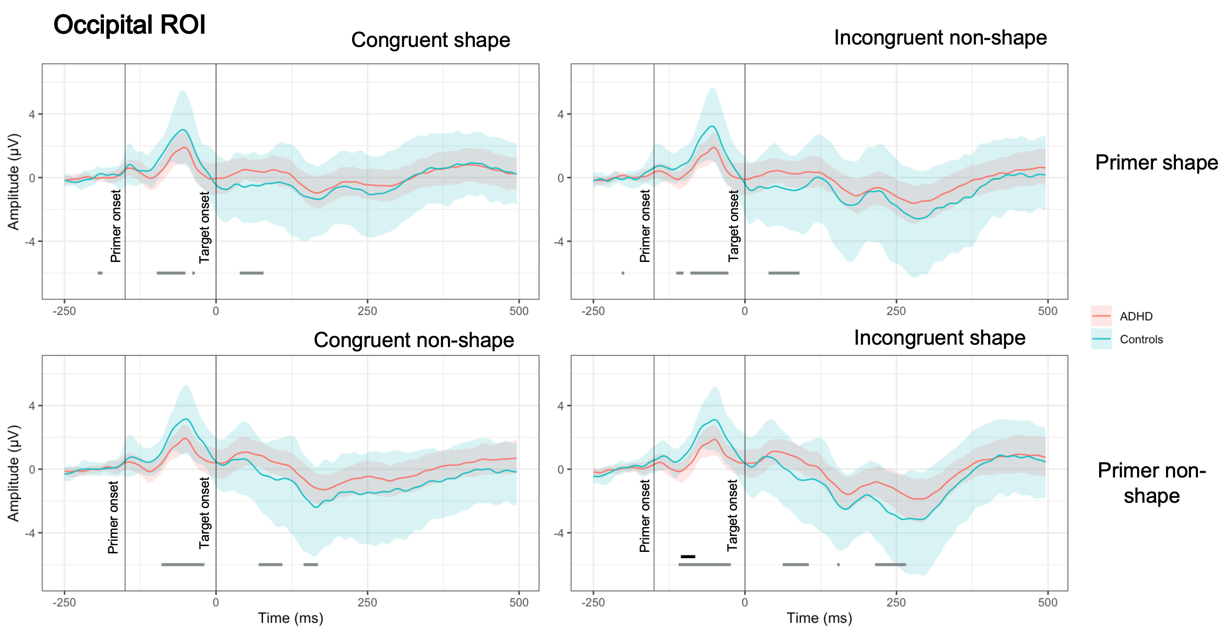


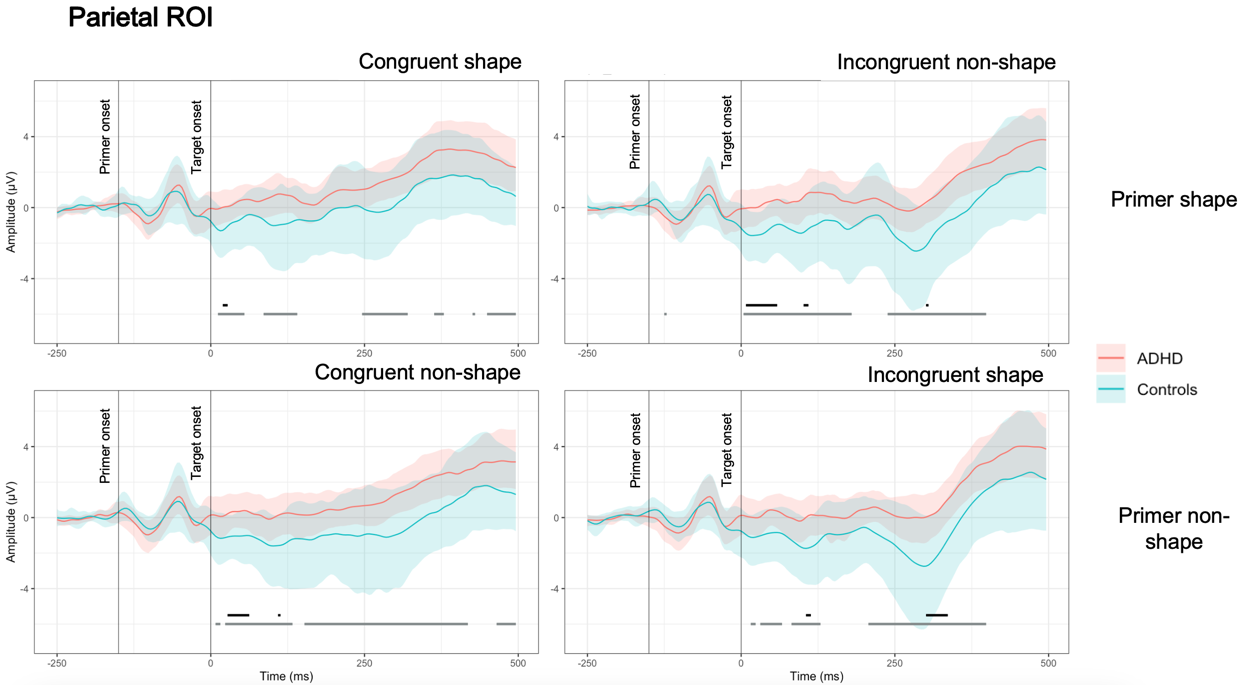


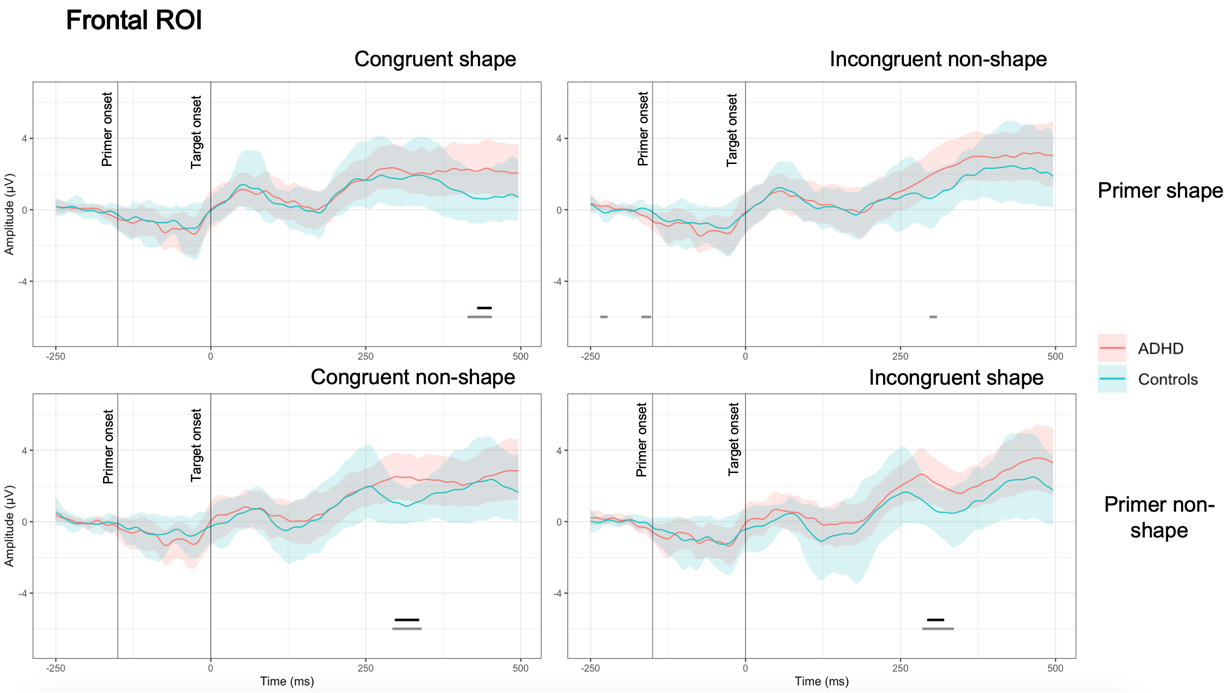


*Supplementary Figure.* Spatial and temporal activations shown by grand average ERPs in each experimental condition in occipital, parietal and frontal ROIs. Each plot shows the average ERPs responses (solid lines) of the ADHD (red) and Control (blue) groups. The mean responses are surrounded by the MWE confidence bands of the time series. The significance lines at the bottom of each plot denote time points where group ERPs were drawn from different distributions (grey: mean of one group is outside MWE of the other group; black: mean of both groups are outside each other's MWE).
